# P05.66 . “It’s a regular part of my schedule, just like brushing my teeth”: the perceived fit of complementary and alternative medicine among young adults

**DOI:** 10.1186/1472-6882-12-S1-P426

**Published:** 2012-06-12

**Authors:** F Sirois

**Affiliations:** 1 Bishop's University, Sherbrooke, Quebec, Canada

## Purpose

As CAM use continues to grow a new generation of CAM consumers are including CAM as part of their repertoire of activities to promote their health and well-being. Yet little is known about how CAM is perceived to fit in with other health-related activities in this population. Understanding how young adult CAM consumers integrate CAM into their lifestyle can help guide policy and practice regarding the needs of this group of CAM consumers. The aim of this mixed methods study was to explore the perceived role of CAM in maintaining and promoting health and well-being among a sample of young and healthy adults.

## Methods

Undergraduate students (N = 359, mean age = 21.2, 86 % female) completed a survey about their use of CAM, self-perceptions of being health-minded, and how CAM fit into their health routine. Forty-four percent were currently using one or more CAM.

## Results

CAM consumers (N=159) were significantly more health-minded than non-consumers, t(357) = 3.89. The CAM consumer responses to the open-ended question “Where does CAM use fit in with the things that you do to take care of your health issues, and/or things that you do to maximize your health and wellness?” were inductively tagged using qualitative content analysis and placed into categories reflecting common themes. Several key themes emerged from the responses. CAM was viewed as a means to deal with athletic injuries, to supplement other health promotion activities, to take care of body and mind, to prevent illness, and as a natural and holistic method for promoting health and well-being.

## Conclusion

These findings echo those of previous research indicating a growing role for CAM in promoting health and well-being and further suggest the ways in which this next generation of CAM consumers integrate CAM use into their lifestyle.

